# TO-UGDA: target-oriented unsupervised graph domain adaptation

**DOI:** 10.1038/s41598-024-59890-y

**Published:** 2024-04-22

**Authors:** Zhuo Zeng, Jianyu Xie, Zhijie Yang, Tengfei Ma, Duanbing Chen

**Affiliations:** 1 School of Computer Science and Engineering, University of Electronic Science and Technology of China, Chengdu, 611731 China; 2 Chengdu Union Big Data Tech. Inc., Chengdu, 610041 China; 3 College of Information Science and Engineering, Hunan University, Changsha, 410082 China; 4 Suining Institute of Digital Economy, Suining, 629018 China

**Keywords:** Graph domain adaptation, Invariant feature representation, Meta pseudo-label, Conditional shift, Generalization, Computational science, Computer science, Information technology

## Abstract

Graph domain adaptation (GDA) aims to address the challenge of limited label data in the target graph domain. Existing methods such as UDAGCN, GRADE, DEAL, and COCO for different-level (node-level, graph-level) adaptation tasks exhibit variations in domain feature extraction, and most of them solely rely on representation alignment to transfer label information from a labeled source domain to an unlabeled target domain. However, this approach can be influenced by irrelevant information and usually ignores the conditional shift of the downstream predictor. To effectively address this issue, we introduce a target-oriented unsupervised graph domain adaptive framework for graph adaptation called TO-UGDA. Particularly, domain-invariant feature representations are extracted using graph information bottleneck. The discrepancy between two domains is minimized using an adversarial alignment strategy to obtain a unified feature distribution. Additionally, the meta pseudo-label is introduced to enhance downstream adaptation and improve the model’s generalizability. Through extensive experimentation on real-world graph datasets, it is proved that the proposed framework achieves excellent performance across various node-level and graph-level adaptation tasks.

## Introduction

Graph neural networks (GNNs) typically rely on end-to-end supervision for training, which often demands a large amount of labeled data^1,2^. Manual labeling of graph data^3,4^, especially in the case of protein-protein interaction (PPI) networks^5^, is a time-consuming task. Furthermore, the absence of labels poses a significant challenge in newly-formed graph domains such as subway and aviation networks^6,7^. It is urgent to alleviate the challenge of sparse labels in the target domain by utilizing relevant or similar labeled domain graph data to train the models. However, recent research demonstrates that graph neural networks’ performance degrades when training models rely solely on labeled source data. The reason for this performance discrepancy is that the data used for training (labeled source data) and inference (unlabeled target data) originate from distinct distributions^8,9^. Consequently, training a well-generalized graph neural network model, especially for only source domain labeled data, presents a significant challenge.

In order to deal with this challenge, many scholars^10–13^ adopt the framework of joint learning to reduce the difference between the representation distributions of two domains. The framework of joint learning can effectively improve the accuracy of target domain unlabeled data, but there are still several critical problems:

(1) The adaptive performance of representation alignment is limited by irrelevant feature interference^14–16^. For instance, in social networks, social networks where the distributions of users’ friendships (the input) and their activity patterns (the label) are significantly influenced by the time and location of data collection^17^. In financial networks^18^, the flows of payments between transactions (the input) and the emergence of illicit transactions (the label) exhibit a strong correlation with external contextual factors such as the time of day or market conditions. These external factors can act as confounding variables, hindering the effectiveness of representation alignment methods. (2) Alignment strategies of domain feature design exhibit variations in different-level graph tasks^19–21^. For example, in the field of graph-level biomolecular, enhancing the feature representation of the subgraph functional groups in a molecule that yield its certain properties may provide insights to guide further experiments^22^. In protein-protein interaction (PPI) networks^5^, node pairs of protein are often used for domain feature extraction to explore the interaction principles between two protein nodes. (3) Ignoring the semantic distribution shift of the target domain^23,24^, such as feature scarcity, varying noise, and temporal evolution, can lead to suboptimal performance in graph adaptation tasks. For instance, in citation networks, the distribution of citations and subject areas changes over time^25^, reflecting the evolving nature of academic research. To address this, it’s crucial to incorporate techniques that adapt to such shifts, enabling models to capture the current state of the network more accurately.

In this report, TO-UGDA addresses the challenge of irrelevant feature interference by leveraging the Graph Information Bottleneck (GIB)^26,27^. This innovative approach effectively filters out superfluous information, focusing solely on the most pertinent features for domain adaptation. This is achieved by learning a compressed representation of the graph structure, which captures the crucial patterns for task performance while excluding noise and irrelevant features. Furthermore, TO-UGDA offers a flexible framework that can seamlessly adapt to varying levels of graph tasks. By establishing a specific sub-graph i.i.d. assumption^28^ and incorporating GIB-based adversarial adaptation training, our framework ensures robust alignment of domain features across diverse graph structures and tasks. This ranges from micro-level information in cross-network node classification to macro-level topology in cross-domain graph classification. Additionally, TO-UGDA incorporates meta pseudo-labels^29^, enabling the model to adapt to semantic distribution shifts in the target domain. By extracting self-semantic information from the target domain data, the model becomes more resilient to feature noise and time evolution, leading to enhanced adaptation and generalization capabilities. Experimental results demonstrate the effectiveness of our proposed method, achieving exceptional performance in two different-level graph tasks while exhibiting remarkable stability.

In summary, this report makes the following contributions:

1. From the perspective of the joint probability distribution, we define and explain the adaptation error bounds of the encoder and predictor.
2. We introduce a novel Target-Oriented Unsupervised Graph Domain Adaptation framework (TO-UGDA) that adopts a GIB-based adversarial strategy to align invariant graph feature representations and incorporates meta pseudo-labeling to bridge the gap in downstream semantic conditional adaptation, resulting in a more generalizable model.
3. TO-UGDA outperforms the baseline in adaptation of micro information in cross-network node classification tasks and macro topology information in cross-domain graph classification tasks.

## Related works

### Unsupervised domain adaptation

Unsupervised domain adaptation (UDA), a crucial branch of transfer learning^30^, aims to address the problem of different distributions by minimizing the distribution discrepancy and transferring label knowledge of source domain^31,32^.

In recent years, many researchers have constantly advocated and paid attention to UDA, such as MMD^33^, DANN^34^, CDAN^35^ and TLDA^36^. In this report, we mainly discuss the adversarial-based domain adaptation method^35,37^ used in our framework. The main idea is minimizing the distance between the source and target domain representation to maximize the confusion of the domain discriminator, which forces the graph encoder can share relevant label knowledge and align feature distribution. The pioneering work DANN^34^ uses the generative-adversarial method of GAN^38^ to align two domains. MADA^39^ and CDAN^35^ take the downstream classification probability as the additional condition information to relieve the problem of downstream conditional shift.

However, it is noted that the assumption of independent and identically distributed (i.i.d.) of representation samples, which holds in classic research fields like computer vision^40,41^, natural language processing^42^, and signal processing technology^43^, does not directly apply to graph domain adaptation. In the graph domain, the graph representation depends on neighboring nodes and edges^44^, making it challenging to satisfy the i.i.d. assumption.

### Graph domain adaptation

In recent years, many researchers in the graph field have proposed graph adaptive learning methods to resolve the alignment challenge under the non-i.i.d. assumptions, which can be divided into two different level types, node-level adaptation, and graph-level adaptation.

Node-level adaptation, which can also be considered as a cross-network task involving the alignment of source and target entire connected networks, has been explored in recent years. UDAGCN^10^ introduces the gradient reversal layer to align cross-network node embedding and develops a dual GCN component to ensure the local and global representation consistency of each node and reduce the irrelevant domain feature dependence. GRADE^45^ proposes a novel graph subtree discrepancy to measure the graph distribution shift between source and target networks, reduces irrelevant domain feature messages passing through graph subtrees and establishes constrained generalized error boundaries.

Graph-level adaptation gives rise to an interesting phenomenon where the graph macro topology representation satisfies the assumption of i.i.d. within the intra-domain (between graphs), but not within the node embedding of the graph itself (between nodes in the same graph)^46,47^. Therefore, it is crucial to strike a balance in the multi-player game of graph node representation, intra-domain topology information, and reducing outer-domain discrepancy. To tackle this challenge, DEAL^24^ employs a clever strategy that combines data augmentation with contrastive learning to address the challenging balance issue that arises in multi-party games. Furthermore, it leverages the encoding features of shallow graph neural networks as clustering information, enabling a clear and distinct differentiation between labels in the target domain. This approach not only enhances the extraction of domain topology feature information but also ensures a more robust and effective performance in handling the complexities of multi-party gaming scenarios. COCO^48^ proposes a coupled graph representation learning approach to extract invariant domain topology information and reduce the domain discrepancy by two different feature encoding modules, which incorporates graph representations learned from complementary views for enhanced domain topology information understanding.

These methods effectively relieve the problem of unsupervised graph domain adaptation. Below we briefly introduce the two main methods used in this report: Graph Information Bottleneck (GIB)^26,27^ is a principle used in graph neural networks to balance the complexity and robustness of learned representations. It ensures that the representation captures enough information to perform the task while avoiding irrelevant information that could lead to overfitting and alignment interference. Meta Pseudo-Labels^29^ is a knowledge distillation technique where the model generating pseudo labels for unlabeled data adjusts its predictions based on the performance of another model trained with these labels. This feedback loop refines the pseudo labels, leading to better model performance over time.

## Problem definition and analysis

This section defines two graph adaptation tasks and analyzes the adaptive error bound of the encoder and predictor from the perspective of the joint probability distribution.

### Problem statement

Inspired by previous works on graph domain adaptation^10,45,48,49^, we formally define two different problems of graph domain adaptation in detail.

#### Problem Formulation 1

(Cross-Network Node Adaptation) Given an unlabeled target single network $G^{t}$ and a labeled source network $(G^{s},Y)$, cross-network node adaptation aims to improve the prediction performance of Node-Level task in the target network by using knowledge from the source network.

#### Problem Formulation 2

(Cross-Domain Graph Adaptation) Given an unlabeled target domain dataset $D^{t}$ and a labeled source domain dataset $(D^{s},Y)$, the purpose of cross-domain graph adaptation is to improve the accuracy of Graph-Level property prediction in the target domain dataset by using knowledge from the source domain.

### Adaptation error bound of encoder and predictor

In graph tasks, it is common to utilize classic architecture such as GNN encoder *P*(*X*) and classifier *P*(*Y*|*X*) to model the joint distribution *P*(*X*, *Y*) between data and labels^50^:

1 $$\begin{aligned} P(X,Y)=P({Y}|{X})P(X) \end{aligned}$$

**Figure 1:**
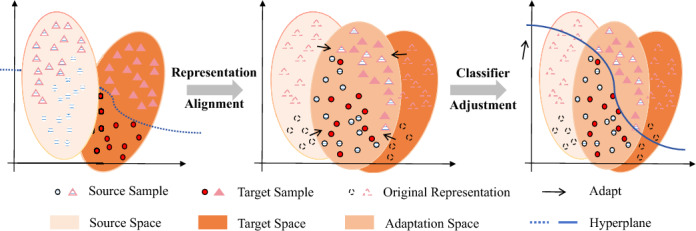
Two main steps of domain adaptation.

Aligning the joint distribution requires two steps, as shown in Fig. 1. The first step is to align the representations of the source domain and target domain data as closely as possible, and the second step is to fine-tune the source domain classifier by extracting the conditional information from the target domain itself.

According to two main steps in Fig. 1, we provide adaptation objective definitions from the perspectives of marginal distribution alignment and conditional distribution alignment.

#### Adaptation Objective 1

(Marginal Distribution Adaptation) Given the source and target graph representations ${H^{s}, H^{t}}$ obtained using the same GNN module with parameter $\theta _f$, margin distribution adaptation refers to minimize the distribution discrepancy of $d\big (P_{s}(x), P_{t}(x)\big )$ of $\{H^{s}, H^{t}\}$, which can be defined as $\underset{\theta _f}{{\text {argmin}}} \Delta _{d}=\underset{\theta _f}{{\text {argmin}}} \int _{-{\infty }}^{+{\infty }} d\big (P_s({x}), P_t({x})\big )dx$.

#### Adaptation Objective 2

(Conditional Distribution Adaptation) Given source and target graph classifiers with parameter $\{\theta _c^{s},\theta _c^{t}\}$, assume conditional distribution $\{P_{s}(y|x),P_{t}(y|x)\}$ of two classifiers can be applied on share representation *P*(*x*), therefore semantic distribution adaptation can be defined as $\underset{\theta _c^{s},\theta _c^{t}}{{\text {argmin}}} \Delta _{d}=\underset{\theta _c^{s},\theta _c^{t}}{{\text {argmin}}} \int _{-{\infty }}^{+{\infty }} d\big (P_{s}(y|x),P_{t}(y|x)\big )P(x)dxdy$.

## Methodology

### Framework

**Figure 2:**
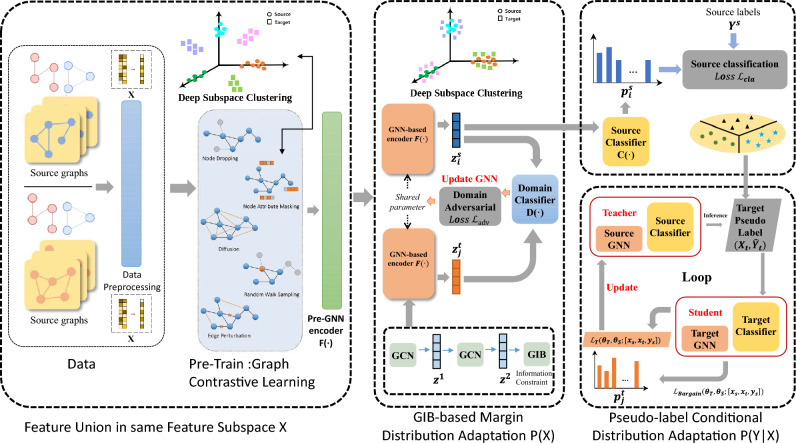
TO-UGDA model architecture includes three main modules: 1) Joint pre-training module for initialization; 2) GIB-based domain adaptation module for aligning invariant features; 3) Meta pseudo-label learning for conditional distribution adaptation.

To optimize these two objectives, there are three key steps in the TO-UGDA training process: (1) Joint pre-training of source and target domain data; (2) GIB-based domain adaptation; (3) Unsupervised meta pseudo-label learning. The model architecture is depicted in Fig. 2.

### Joint pre-training initialization based on contrastive learning

Contrastive pre-training initialization has been proven to be beneficial for various graph tasks^51,52^. By combining data from two domains and applying self-supervised contrastive learning, the GNN encoder $Z=F(x)$ is capable of learning generalized feature embedding and unifying the representation space.

For a given original sample $x_i$, multiple similar disturbance samples ${ x_j }$ constitute a part of the positive pairs, and other samples that are far from the given original sample are constructed as negative pairs. The initialization GNN encoder is trained using a contrastive learning loss function:

2 $$\begin{aligned} \mathcalligra {L}_{con}=-\log _{}{\frac{exp \big ( \frac{sim(z_i,z_j)}{\tau } \big ) }{{\textstyle \sum _{k}^{}exp \big ( \frac{sim(z_i,z_k)}{\tau } \big )} }} \end{aligned}$$

where $sim(\cdot , \cdot )$ denotes cosine similarity between two vectors and $\tau$ is a temperature parameter. This loss function encourages the embeddings of the positive pair $(x_i, x_j^+)$ to be close to each other while pushing the embeddings of the negative pair $(x_i, x_j^-)$ further apart.

### GIB-based invariant representation domain adaptation

Graph embedding violates the i.i.d. assumption, posing an alignment challenge for acquiring invariant information due to the node representation dependence on their neighboring nodes. Therefore, TO-UGDA needs to design a special encoder to extract invariant information, and then build GIB-based domain adaptation.

#### Invariant graph representation

As assumed by information theory^26,28^, node representations can be locally dependent on their important neighboring structures. Therefore, we establish a specific i.i.d. assumption that local neighborhood structure can represent each node in the graph, which enables it to adopt representation learning based on mutual information to extract invariant features.

In this report, we extract crucial neighborhood structural information from the original graph structure, denoted as $A_v^{(l)}$ and described by a Bernoulli distribution with parameter $\alpha _{v}^{(l)} \in [0, 1]$. This information is obtained to update the node representation $Z_v^{(l)}\in R^{n}$ using the *l*-th layer GNN with parameter $W^{(l)}$, as detailed below:

3 $$\begin{aligned} \left\{ \begin{array}{c} \alpha _{v}^{(l)} \leftarrow {\text {Sigmoid}}\left( \left\{ \left( Z_v^{(l-1)} \oplus Z_{u_1}^{(l-1)}\oplus \cdots \oplus Z_{u_{N_v}}^{(l-1)}\right) \right\} _{u_1,\cdots ,u_{N_v} \in G_v}\right) \\ A_v^{(l)}\leftarrow \left\{ \begin{array}{c} u \in G_v^l \mid u \overset{iid}{\sim }\ {\text {Bernoulli}}\left( \alpha _{v}^{(l)}\right) \\ \end{array}\right\}. \\ Z_v^{(l)}\leftarrow {\text {ReLU}}\big ( {D}^{-\frac{1}{2}}{(A_v^{(l)}+I)}{D}^{-\frac{1}{2}}Z_v^{(l-1)}W^{(l)} \big ) \end{array}\right. \end{aligned}$$

where, $N_v$ represents the node number about neighborhood structure $G_v$ of node *v*. Furthermore, the graph-level representation utilizes a ${\text {Readout}}(\cdot )$ function to represent each graph in the dataset, which is defined as:

4 $$\begin{aligned} Z_G \leftarrow {\text {Readout}}\left( \left\{ Z_{u_1}^{(l)} \oplus \cdots \oplus Z_{u_{n}}^{(l)} \right\} _{u_1,\cdots u_{n} \in G}\right) \end{aligned}$$

where $Z_G\in R^{n}$ is the *n*-dimensional invariant feature representation of input samples.

#### GIB-based domain adaptation

Inspired by information bottleneck theory^26,53^, the adaptation module of TO-UGDA is encouraged to maximize the mutual information between the source domain representation $Z_s$ and the label $Y_s$ to enhance prediction accuracy on source labeled data $(X_s,Y_s)$, and maximize the mutual information between two domains representation $(Z_s,Z_t)$ to align domain distribution. Finally, the graph information bottleneck avoids the interference of excessive irrelevant information. Therefore, the multi-objective optimization can be defined as:

5 $$\begin{aligned} \begin{aligned} \max _{\mathbb {P}\left( Z|X\right) \in \Omega } I\left( Z_s ; Y_s\right) +I\left( Z_s ; Z_t\right) \\ \text{ s.t. } I^{S}\left( X_s ; Z_s\right) \le \gamma , I^{T}\left( X_t ; Z_t\right) \le \gamma \end{aligned} \end{aligned}$$

where, $\Omega$ is the search space of the optimal representation model $\mathbb {P} (Z|X)$, $I(\cdot ;\cdot )$ denotes the mutual information, $I^{S}(X_s; Z_s) \le \gamma$ and $I^{T}(X_t; Z_t) \le \gamma$ act as double GIBs constraints enable to limit the propagation of both domains information between the original input samples *X* and their invariant feature representation *Z*.

Due to the high computational complexity of mutual information measurement in the calculation process of constraint term, the variational upper bound $I^{up}(X; Z)$ is used to effectively implement double GIBs information constraints about $I^{S}(X_s; Z_s)$ and $I^{T}(X_t; Z_t)$, and the proof is detailed in *Supplementary Appendix A*.

The first objective term $\max I\left( Z_s; Y_s\right)$ in Eq. (5) can be equivalently achieved by minimizing the classification loss $\mathcalligra {L}_{c l a}\left( F, C ; \theta _{f, c}\right)$ for the representation *Z* about the graph data *G* via invariant sub-information $G_{sub}$, as follows:

6 $$\begin{aligned} \begin{aligned} \max I\left( Z_{s} ; Y_s\right)&\overset{iid}{=}\ \max I\left( G_{sub} ; Y\right) = \max \mathbb {E}_{G_{sub}, Y}\left[ \log \frac{\mathbb {P}\left( Y \mid G_{sub}\right) }{\mathbb {P}\left( Y\right) }\right] \\ {}&\ge \max \mathbb {E}_{G_{sub}, Y}\left[ \log \frac{\mathbb {P}_{\theta }\left( Y \mid G_{sub}\right) }{\mathbb {P}\left( Y\right) }\right] = \max KL\left( \mathbb {P}_{data}\left( Y \right) || \mathbb {P}_{\theta }\left( Y \mid G_{sub}\right) \right) = \min \mathcalligra {L}_{c l a}\left( F, C ; \theta _{f, c}\right) \end{aligned} \end{aligned}$$

where, $\mathbb {P}_{\theta }\left( Y \mid G_{sub}\right)$ is a variational approximation of $\mathbb {P}\left( Y \mid G_{sub}\right)$ to solve the intractable challenge of $\mathbb {P}\left( Y \mid G_{sub}\right)$, the proof is detailed in *Supplementary Appendix B1 and B2*.

Meanwhile, the second objective term $\max I\left( Z_s; Z_t\right)$ in Eq. (5) can be equivalently achieved by the adversarial loss $\mathcalligra {L}_{a d v}\left( F, D ; \theta _{f, d}\right)$ of the discriminator *D* to maximize the lower-bound *Donsker-Varadhan Representation*^54,55^, as follows:

7 $$\begin{aligned} \begin{aligned} \max I\left( Z_s ; Z_t\right)&= \max \mathbb {E}_{Z_s, Z_t}\left[ \log \frac{\mathbb {P}\left( Z_s , Z_t\right) }{\mathbb {P}\left( Z_s\right) \mathbb {P}\left( Z_t\right) }\right] = \max \mathbb {E}_{s,t}\Big [ KL\Big (\mathbb {P}\left( Z_s , Z_t\right) || \mathbb {P}\left( Z_s\right) \mathbb {P}\left( Z_t\right) \Big ) \Big ] \\&\ge \max I_{\theta }^{DV} \left( Z_s ; Z_t\right) = {\max } I_{\theta }^{DV} \big (F(x_s),F(x_t)\big )\\ {}&= \max \mathbb {E}_{\mathbb {P}\left( Z_s,Z_t\right) }[{D_{\theta }(F(x_s),F(x_t))}] - \log ({\mathbb {E}_{\mathbb {P}\left( Z_s\right) \mathbb {P}\left( Z_t\right) }[e^{D_{\theta }(F(x_s),F(x_t))}])} \\&= \max \mathcalligra {L}_{adv}\left( F, D ; \theta _{f, d}\right) \end{aligned} \end{aligned}$$

where, $\mathcalligra {L}_{adv}\left( F, D ; \theta _{f, d}\right)$ is an instance $D_{\theta }$ of any class $\mathcalligra {F}$ of function $T:\Omega \rightarrow \mathbb {R}$, which satisfying the integrability constraints of the *Donsker-Varadhan Representation* by a deep neural network with parameter $\theta \in \Theta$ to obtain the lower-bound *Donsker-Varadhan Representation* $I_{\theta }^{DV} \left( Z_s; Z_t\right)$, the proof is detailed in *Supplementary Appendix B3*.

Each mutual information term in Eq. (5) can be efficiently calculated, therefore, the final adaptation optimization loss function is:

8 $$\begin{aligned} \begin{aligned} \mathcalligra {L}_{\text{ adapt } }\left( F,C,D \right) = \underset{F,C}{{\text {min}}} \ \underset{D}{{\text {max}}} \Big ({\mathcalligra {L}_{\text{ cla }}\left( F,C;\theta _{f,c} \right) + \mathcalligra {L}_{\text{ adv }} ( F,D;\theta _{f,d} ) + \beta \big ( I^{\text{ up }}_{\theta _{f}}\left( X_s;Z_s \right) + I^{\text{ up }}_{\theta _{f}}\left( X_s;Z_t \right) \big )} \Big ) \end{aligned} \end{aligned}$$

where $\beta$ is the weight factor about invariant representation, and the Eq. (8) is derived from Eq. (5) by GIB paradigm^26^ and Lagrange multiplier approach, the proof is detailed in *Supplementary Appendix B4*.

The GIB-based domain adaptation ensures that only the invariant features of two domains can be aligned to the same representation distribution and transfer the label information *Y*.

### Unsupervised meta pseudo-label distillation

Inspired by meta pseudo-label knowledge distillation^29^, the teacher model actively participates in Boundary Bargaining Game (a term referring to the process of refining decision boundaries) and knowledge propagation on unlabeled data. Meanwhile, the student’s performance, which feeds back on labeled data testing after pseudo-label distillation from the teacher model, influences the direction and weight of the boundary games in the teacher model’s next step. This balance ensures both the generalization of unlabeled data and the fitting of labeled data.

In our work, we also consider target domain as the most crucial aspect, that the approach effectively reduces the discrepancy of conditional distribution adaptation $\int _{-{\infty }}^{+{\infty }} d\big (P_{s}(y|x),P_{t}(y|x)\big )P(x)dxdy$ about the self-semantic information of target domain and the transfer knowledge of the source domain by the student testing performance. Furthermore, it alleviates the limitation of current graph adaptation methods, which often overemphasize source-labeled data and neglect target domain semantic conditional information. However, the most crucial step here is how the teacher model updates based on the performance of the student model.

Let *T* and *S* denote the teacher model and the student model, respectively, parameterized by $\theta _T$ and $\theta _S$. The ultimate training objective of TO-UGDA lies in achieving Bargaining Game’s Nash equilibrium between the self-semantic information of the target domain and the transfer knowledge of the source domain, by quantifying the classification loss of the student $\theta _S$ on unseen true labeled source domain data:

9 $$\begin{aligned} \underset{\theta _S,\theta _T}{\min }\mathcalligra {L}_{\text{ cla }}\bigg (S\big (\left[ x_s\right] ; \theta _S(\theta _T)\big ), y_s\bigg ) \end{aligned}$$

where $\theta _S(\theta _T)$ represents the relationship that student $\theta _S$ rely on the pseudo-label generated by teacher $\theta _T$.

During the bargaining distillation process, the student model is trained using pseudo labels generated by the teacher model in the target domain, and the teacher model is updated based on the student’s test performance on unseen true labeled source domain data. However, it is a challenge to directly update the teacher model’s parameters and achieve the Nash equilibrium of the bargaining distillation process by the performance of the student model. This process involves three key steps, as follows:

(1) Training the teacher model using labeled source domain dataset and unlabeled target domain dataset, with the optimization objective for $\theta _T$ can be defined as:

10 $$\begin{aligned} \theta _T^{'}=\underset{\theta _T}{{\text {argmin}}} \mathcalligra {L}_{\text{ adapt } }\big (T\left( \left[ x_s, x_t\right] ; \theta _T\right) , y_s\big ) \end{aligned}$$

where $\mathcalligra {L}_{\text{ adapt }}$ is the adaptation loss function presented in Eq. (8).

(2) Training the student model $\theta _S$ using the pseudo labels $(x_t, {\hat{y}}_t)$ generated by the teacher model $\theta _T$, the optimization objective is:

11 $$\begin{aligned} \theta _S^{'}=\underset{\theta _S}{{\text {argmin}}} \mathcalligra {L}_{\text{ adapt } }\bigg (S\big (\left[ x_s, x_t\right] ; \theta _S\big ), {\hat{y}}_t\bigg ) \end{aligned}$$

where student model $\theta _S$ is initialized based on unsupervised semantic clustering.

(3) Obtaining the bargaining distillation loss $\mathcalligra {L}_{\text{ distill }}(T,S;[x_s, x_t, y_s])$, which is used to correct the updating direction of the teacher model based on the performance of the student model’s parameters, as follows:

12 $$\begin{aligned} \begin{aligned} \mathcalligra {L}_{\text{ distill }}(T,S;[x_s, x_t, y_s])=\left( \mathcalligra {L}_{\text{ cla }}\left( S\left( x_t ; \theta _S\right) , y_s\right) -\mathcalligra {L}_{\text{ cla } }\left( S\left( x_s ; \theta _S^{'}\right) , y_s\right) \right) \cdot \mathcalligra {L}_{\text{ adapt } }\big (T\left( \left[ x_s, x_t\right] ; \theta _T\right) , {\hat{y}}_t\big ) \end{aligned} \end{aligned}$$

where $\mathcalligra {L}_{\text{ distill }}$ is expressed as the product of two derivatives (the student testing performance of pseudo-label and the teacher adaptation of soft pseudo-label), the details of the proof are described in *Supplementary Appendix C1*. Therefore, the updated adaptation loss function of teacher model is:

13 $$\begin{aligned} \begin{aligned} \mathcalligra {L}_{\text{ teacher }}(T,S;[x_s, x_t, y_s]) = \mathcalligra {L}_{\text{ adapt } }\big (T\left( \left[ x_s, x_t\right] ; \theta _T\right) , y_s\big ) + \mathcalligra {L}_{\text{ distill }}(T,S;[x_s, x_t, y_s]) \end{aligned} \end{aligned}$$

In the learning process, the student model can improve the adaptive ability of the overall model in the target domain, by leveraging target domain self-semantic information to limit the parameter search space of the teacher model. This method is helpful to improve the accuracy and efficiency of target domain prediction. Specifically, we describe the training algorithm of TO-UGDA in the * Supplementary Appendix C2*.

## Experiments and analysis

### Datasets

The effectiveness of the method is evaluated on multiple adaptation datasets with varying cause types, demonstrating its generalized adaptability. Detailed statistics are presented in Tables 1 and 2. In addition, we present the results of experiments on cross-network (node-level) and cross-domain (graph-level) adaptation tasks.

#### Cross-network

1. **Air-Traffic Network**^56^: The dataset comprises air traffic networks in the United States, Europe, and Brazil, where each node represents an airport and an edge indicates the presence of commercial flights. The categories of airports are determined based on building size and aircraft activity.
2. **Citation Networks**^57^: The citation networks DBLPv8 and ACMv9 are two paper citation networks. Each edge in these networks represents the citation relationship between two papers, where each node represents a paper and the class label indicates the research topic.

#### Cross-domain

In our experiment, we utilized various real-world datasets from different research areas and backgrounds in TUDataset^58^ to compare the performance of different baselines.

1. **Mutagenicity**: The mutagenicity dataset comprises molecular structures and Ames test data. We divide the molecular structures into four different distribution sub-datasets (namely M0, M1, M2, and M3) based on the edge density of these structures.
2. **Letter-Drawings**: This dataset consists of distorted letter drawings, as well as their variations at different intensity levels (low, medium, and high). For each class, multiple prototype drawings are manually constructed by using undirected edges and nodes to represent the handwritten alphabet.
3. **NCI**: A biological dataset for the classification of anticancer activities. In this dataset, each graph represents a chemical compound, where nodes and edges represent atoms and chemical bonds, respectively. NCI1 is an activity screening for non-small cell lung cancer cells, and NCI109 is an activity screening for ovarian cancer cells.

**Table 1:** Statistics of cross-network datasets.

Dataset	#Nodes	#Edges	#Features	#Labels
Brazil	131	1038	–	4
Europe	399	5995	–	4
USA	1190	13599	–	4
DBLPv8	5578	7341	7537	6
ACMv9	7410	11135	7537	6

**Table 2:** Statistics of cross-domain datasets.

Dataset	#Size	#Avg nodes	#Avg edges	#Labels
Mutagenicity	4337	30.32	30.77	2
Letter-low	2250	4.68	3.14	15
Letter-med	2250	4.67	3.21	15
Letter-high	2250	4.67	4.50	15
NCI1	4110	29.87	32.30	2
NCI109	4127	29.68	32.13	2

### Baselines

Three type baseline models are used for cross-network node classification adaptation: (1) Source-Only: GCN^59^, SGC^60^, GCNII^61^; (2) Node Feature-Only adaptation: CDAN^35^, DANN^62^, MDD^63^; (3) cross-network adaptation: AdaGCN^64^, UDAGCN^10^, EGI^49^, and GRADE^45^. For cross-domain graph classification adaptation, the following three type baselines are used to compare: (1) Source-Only: GCN^59^, SGC^60^, GIN^44^, (2) Traditional domain adaptation methods: CDAN^35^, ToAlign^65^ and MetaAlign^66^, whose feature encoder is replaced with GCN. (3) Graph cross-domain adaptation: DEAL^24^ and COCO^48^, a customized framework for adaptation tasks of graph classification.

### Implementation details

We adopt a two-hidden-layer graph convolutional network as the feature extractor and a single layer of fully connected neural networks. In addition, the teacher model and the student model are optimized using SGD and Adam optimizers, with learning rates of 0.02 and 0.001. Each experimental result is the mean value through three repetitions and 200 epochs. We use Accuracy(ACC) as the classification metric.

### Performance comparison

#### Cross-network

We conducted extensive experiments on the Air-Traffic Networks and Citation Networks. The experiment results of all methods in node classification are shown in Tables 3 and 4. From Tables 3 and 4, it can be seen that (1) Node Feature-Only adaptation performs worse than the Source-Only method in Citation Network. This can be attributed to the presence of an obvious community structure and topic citation style in Citation Network. (2) Cross-network adaptation achieves the best performance in node adaptation classification compared to other baselines. The reason is that the cross-network adaptation method can simultaneously align node features and topological structure information, effectively enhancing the model’s adaptability to graph-structured data. (3) TO-UGDA outperforms all other methods in node adaptation classification. Specifically, TO-UGDA achieves improvements of 3.8% to 14.5%, and 6.5% to 25.3% on the Air-Traffic and Citation networks, respectively.

**Table 3:** Cross-network node classification on the Airport network.

Methods	USA$\rightarrow$Brazil	USA$\rightarrow$Europe	Brazil$\rightarrow$USA	Brazil$\rightarrow$Europe	Europe$\rightarrow$USA	Europe$\rightarrow$Brazil	Avg.
GCN^59^	0.366	0.371	0.491	0.452	0.439	0.298	0.403
SGC^60^	0.527	0.430	0.432	0.479	0.447	0.481	0.466
GCNII^61^	0.344	0.393	0.470	0.494	0.460	0.542	0.450
CDAN^35^	0.511	0.389	0.441	0.398	0.435	0.539	0.452
DANN^62^	0.500	0.386	0.402	0.350	0.436	0.538	0.435
MDD^63^	0.500	0.378	0.402	0.350	0.402	0.477	0.418
AdaGCN^64^	0.466	0.434	0.501	0.486	0.456	0.561	0.484
UDAGCN^10^	0.607	0.388	0.497	0.510	0.434	0.477	0.486
EGI^49^	0.523	0.451	0.417	0.454	0.452	0.588	0.481
GRADE^45^	0.550	0.457	0.497	0.506	0.463	0.588	0.510
TO-UGDA	0.725	0.521	0.463	0.531	0.474	0.565	0.548

**Table 4:** Cross-network node classification on the citation network.

Methods	ACM$\rightarrow$DBLP	DBLP$\rightarrow$ACM	Avg.
GCN^59^	0.435	0.567	0.501
SGC^59^	0.430	0.611	0.520
GCNII^61^	0.465	0.559	0.512
CDAN^35^	0.342	0.434	0.388
DANN^62^	0.368	0.381	0.374
MDD^63^	0.349	0.391	0.370
AdaGCN^64^	0.451	0.566	0.508
UDAGCN^10^	0.516	0.600	0.558
EGI^49^	0.489	0.404	0.446
GRADE^45^	0.475	0.635	0.555
TO-UGDA	0.582	0.664	0.623

#### Cross-domain

Cross-domain adaptation is a multi-graph alignment problem, resulting in diverse application scenarios for adaptation. We conducted experiments on three representative datasets: Mutagenesis (for edge density adaptation), Letter-drawing (for noise interference adaptation), and NCI (for label application adaptation).

The experimental results of all methods in graph classification are shown in Tables 5, 6, and 7. Several observations need to be highlighted: (1) Traditional domain adaptation methods did not show performance improvement, compared to the Source-Only method in three graph domain datasets. This is because cross-domain adaptation is susceptible to noise affecting the graph structure and irrelevant feature information from neighboring nodes. (2) Graph cross-domain adaptation outperforms other baselines by incorporating shallow representation semantic information and topological structure alignment. (3) TO-UGDA outperforms all compared methods in graph adaptation classification. Specifically, TO-UGDA achieves improvements of 0.2% to 18.9%, 3.8% to 18.92%, and 3.26% to 11.06% on the Mutagenicity, Letter-Drawing and NCI datasets, respectively. These significant breakthroughs can be attributed to the incorporation of GIB-based domain adversarial learning and pseudo-label knowledge distillation, making our model more generalized and adaptable to diverse adaptation scenarios.

**Table 5:** Cross-domain graph classification on Mutagenicity dataset.

Methods	M0$\rightarrow$M1	M1$\rightarrow$M0	M0$\rightarrow$M2	M2$\rightarrow$M0	M0$\rightarrow$M3	M3$\rightarrow$M0	M1$\rightarrow$M2	M2$\rightarrow$M1	M1$\rightarrow$M3	M3$\rightarrow$M1	M2$\rightarrow$M3	M3$\rightarrow$M2	Avg.
GCN^59^	0.711	0.704	0.627	0.690	0.577	0.596	0.688	0.742	0.536	0.633	0.658	0.745	0.659
SGC^60^	0.730	0.695	0.636	0.704	0.543	0.603	0.682	0.737	0.542	0.614	0.593	0.724	0.650
GIN^44^	0.723	0.685	0.641	0.721	0.566	0.611	0.674	0.744	0.559	0.673	0.628	0.730	0.663
CDAN^35^	0.738	0.741	0.689	0.714	0.579	0.596	0.700	0.741	0.604	0.671	0.592	0.636	0.667
ToAlign^65^	0.740	0.727	0.691	0.652	0.547	0.731	0.717	0.772	0.587	0.731	0.615	0.622	0.678
MetaAlign^66^	0.667	0.514	0.570	0.514	0.464	0.514	0.570	0.667	0.464	0.667	0.464	0.570	0.554
DEAL^24^	0.763	0.726	0.698	0.733	0.583	0.712	0.779	0.808	0.641	0.741	0.706	0.749	0.720
COCO^48^	0.777	0.766	0.733	0.745	0.666	0.743	0.773	0.808	0.674	0.741	0.689	0.775	0.741
TO-UGDA	0.786	0.757	0.731	0.757	0.612	0.623	0.803	0.835	0.797	0.733	0.727	0.756	0.743

**Table 6:** Cross-domain graph classification on Letter-Drawing dataset.

Methods	Low$\rightarrow$Med	Low$\rightarrow$High	Med$\rightarrow$Low	Med$\rightarrow$High	High$\rightarrow$Low	High$\rightarrow$Med	Avg.
GCN^59^	0.4822	0.2982	0.6382	0.2800	0.5391	0.3884	0.4377
SGC^60^	0.5253	0.2160	0.7921	0.2908	0.5346	0.3986	0.4596
GIN^44^	0.4973	0.2102	0.7812	0.2849	0.5049	0.4102	0.4481
CDAN^35^	0.4012	0.2367	0.6587	0.2154	0.5071	0.4483	0.4112
ToAlign^65^	0.5829	0.2570	0.6374	0.2714	0.5946	0.4768	0.4700
MetaAlign^66^	0.5271	0.2638	0.6819	0.2932	0.5623	0.5122	0.4734
DEAL^24^	0.5731	0.3189	0.7413	0.2911	0.6136	0.5587	0.5161
COCO^48^	0.5965	0.3347	0.7893	0.3149	0.7445	0.5947	0.5624
TO-UGDA	0.6212	0.3473	0.8526	0.3355	0.8318	0.6139	0.6004

**Table 7:** Cross-domain graph classification on NCI.

Methods	NCI1$\rightarrow$NCI109	NCI109$\rightarrow$NCI1	Avg.
GCN^59^	0.6472	0.6377	0.6424
SGC^60^	0.6055	0.6292	0.6173
GIN^44^	0.6603	0.6493	0.6548
CDAN^35^	0.6953	0.6134	0.6543
ToAlign^65^	0.6764	0.6516	0.6640
MetaAlign^66^	0.6938	0.6412	0.6675
DEAL^24^	0.7129	0.6202	0.6665
COCO^48^	0.7326	0.6581	0.6953
TO-UGDA	0.7569	0.6990	0.7279

### Ablation study

We conduct an ablation study and analysis using citation networks as an example. In this study, we selectively remove components of TO-UGDA: pre-training with joint contrastive learning (Pre-Training), GIB-based domain adversarial adaptation (GIBDA), and pseudo-label knowledge distillation (Distill). This process results in six different variant models (A-F).

**Figure 3:**
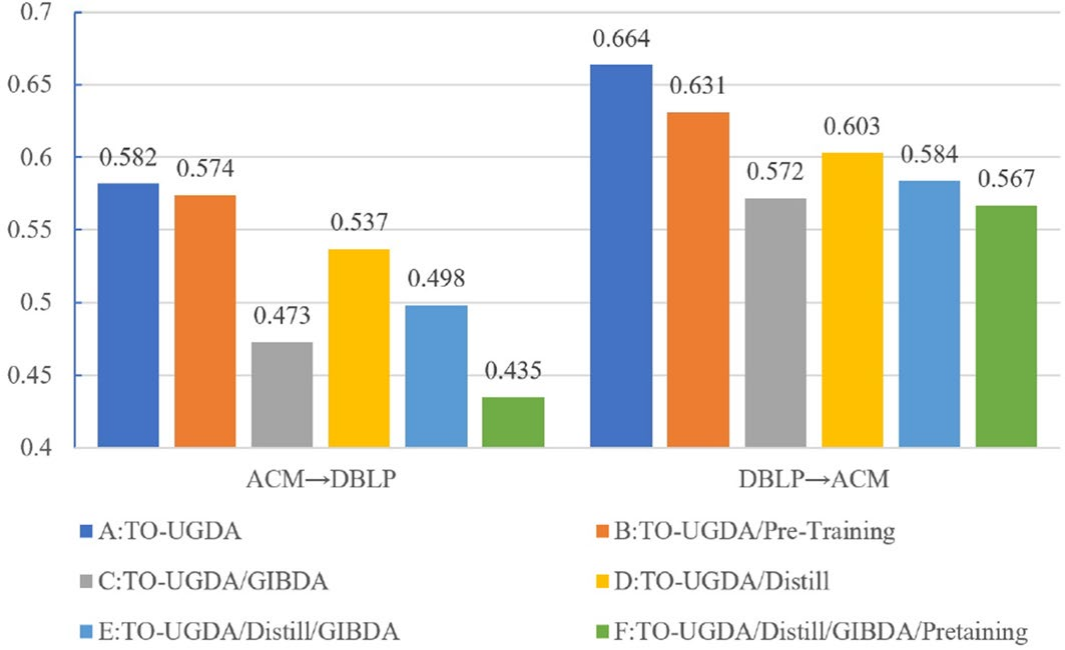
The results of ablation studies on Citation Dataset, ‘/’ represents the removal of the module.

The ablation results are shown in Fig. 3, and we can obtain several observations. (1) The complete TO-UGDA outperforms all other variant models, which validates the importance of each module in unsupervised graph adaptation. (2) The score of variant C rapidly drops by 10.9% and 9.2% when the GIBDA component is removed, empirically validating the significance of invariant feature alignment. (3) Compared to variant D, the removal of the Distill component caused a decrease of 4.5% and 5.1%. This demonstrates that pseudo-label knowledge distillation effectively mines latent target-oriented information. (4) Comparing variants C and E, after removing GIBDA, the existence of the Distill module still reduces the accuracy by 2.5% and 1.2%, indicating that the latent information mining of Distill relies on the invariant feature filter and alignment.

### Training stability evaluation and adaptation weight analysis

**Figure 4:**
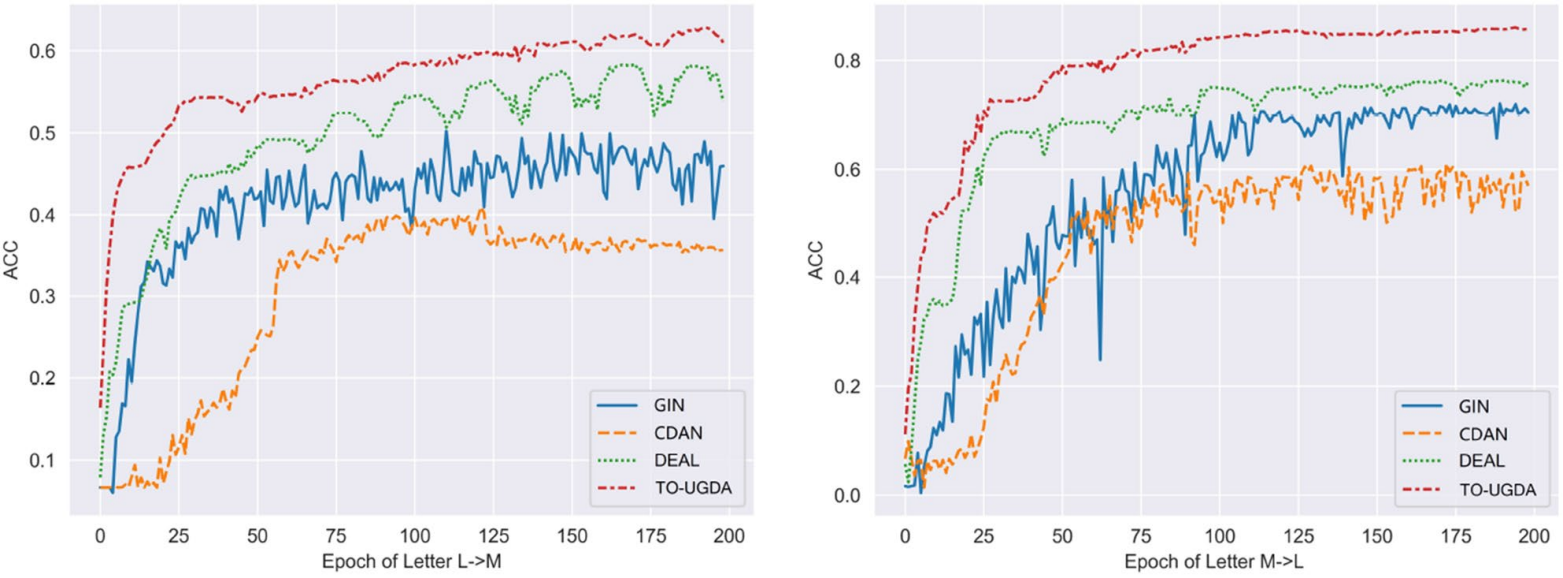
Training stability (Left:L$\rightarrow$M; Right:M$\rightarrow$L).

Models with weak adaptive ability are prone to exhibiting significant fluctuations in target domain accuracy, both before and after each round of parameter updates. Furthermore, our method exhibits superior convergence performance compared to other methods, even in early training iterations, as depicted in Fig. 4. This demonstrates the effectiveness of our approach, which benefits from the initialization of joint pre-training, enabling faster convergence and reduced training costs.

**Figure 5:**
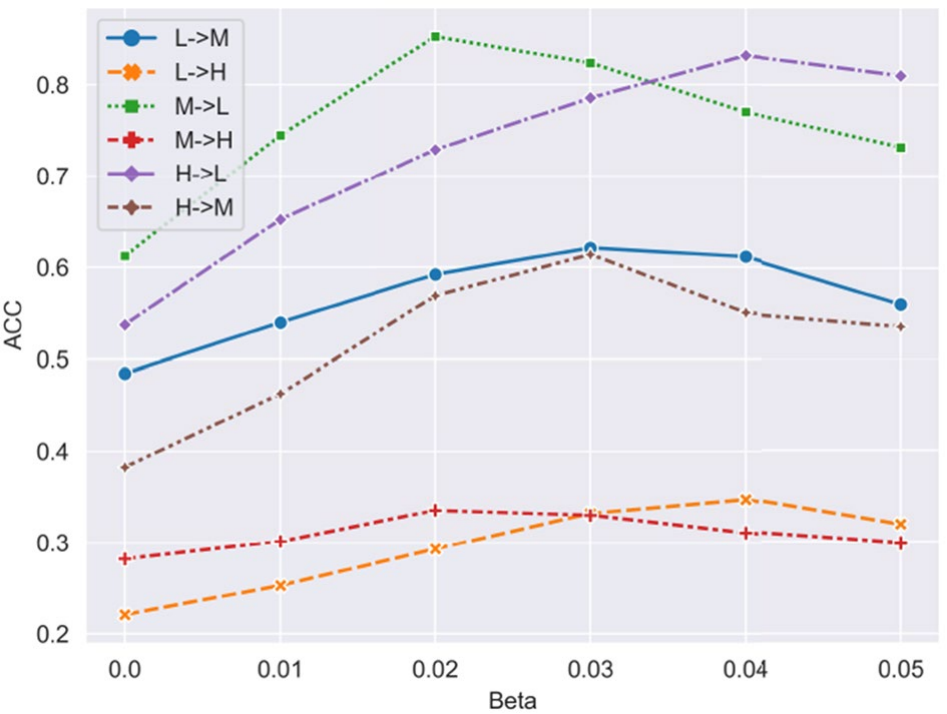
Adaptation weight analysis.

Additionally, in Fig. 5, we observed that the performance of TO-UGDA initially increases and then decreases as the adaptation weight parameter $\beta$ varies from 0 to 0.05. This phenomenon occurs because a small weight for GIBDA fails to provide sufficient graph adaptation and invariant feature representation ability, while a large weight misleads the objective function, neglecting the learning of source domain features and labels.

### Visualization of T-SNE

**Figure 6:**
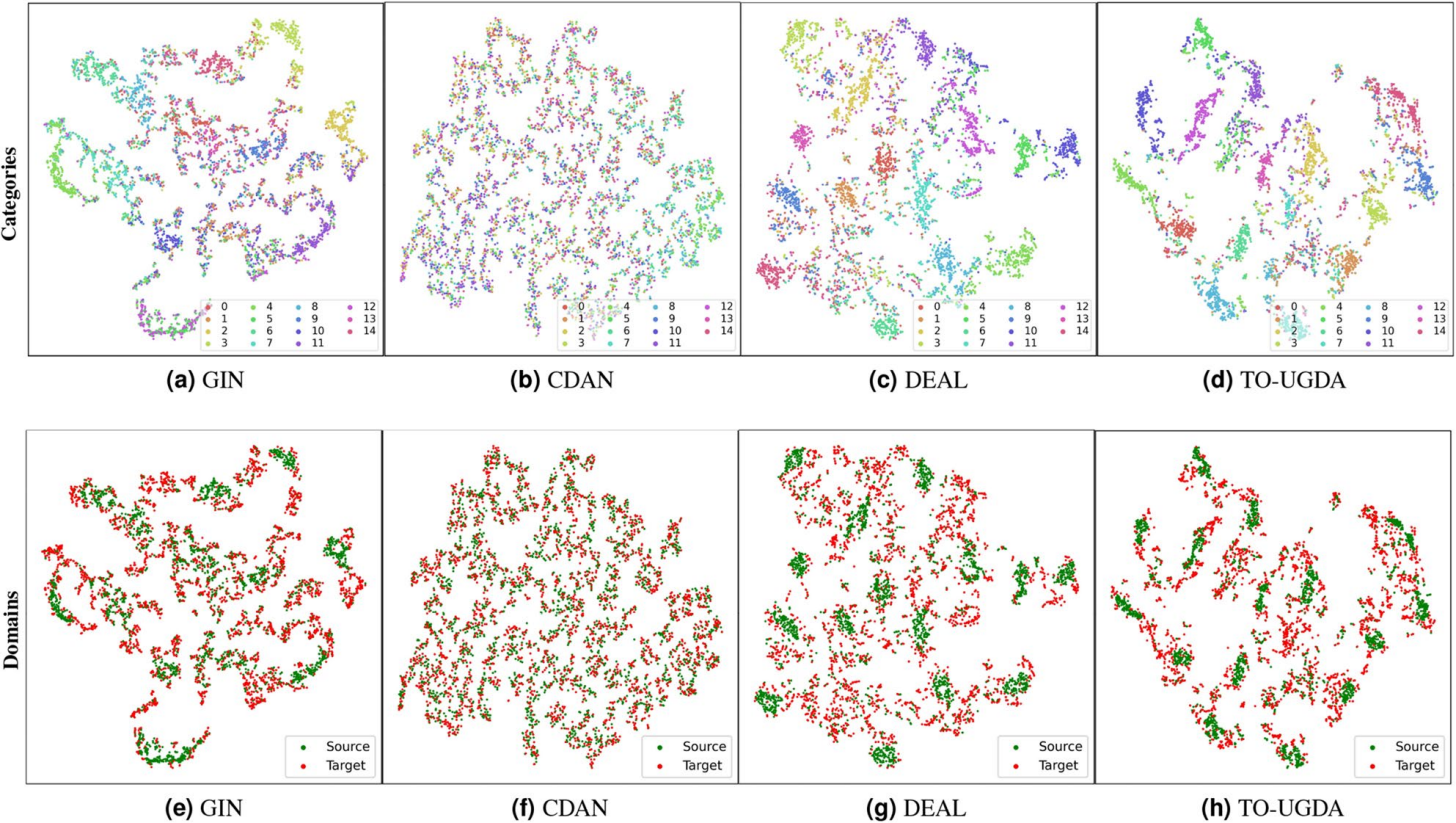
Source(Letter-Low)$\rightarrow$Target(Letter-Med): Visualize the extracted features through T-SNE. The colors in the first row represent different categories, while the colors in the second row represent different domains.

The visualization of the graph representations learned from our method and other baselines has been presented in Fig. 6. We observed that (1) Traditional adversarial domain adaptive method CDAN overly focus on complete feature distribution alignment, causing significant alignment interference by irrelevant features, as shown in Figs. 6b and f. (2) The representation distribution of TO-UGDA exhibits better local clustering and global separation in classification than GIN and DEAL in Fig. 6d. The source domain and target domain data distributions exhibit good alignment performance in Fig. 6h.

## Conclusions and future work

In conclusion, we have defined the adaptive error bounds of the encoder and predictor, explaining them from the perspective of joint distribution probability. Drawing inspiration from this analysis, we propose TO-UGDA, a novel graph domain adaptation framework that leverages invariant feature alignment to extract essential information while discarding irrelevant details. TO-UGDA effectively addresses the challenges of Target-Oriented Unsupervised Graph Domain Adaptation. Extensive experimentation has demonstrated the superior performance of TO-UGDA over all baselines. The experimental outcomes demonstrate that by aligning invariant features, our model can effectively capture shared invariant features between the source and target domains. These invariant features remain consistent across different domains, enabling the model to seamlessly adapt to novel and unprecedented data, thereby enhancing its generalization capabilities. Furthermore, by emphasizing these invariant features, our approach minimizes the negative transfer effects that often arise due to domain discrepancies. Additionally, incorporating semantic information into the target domain further aids the model in grasping the intricate relationships between transferred knowledge and the inherent structure and meaning of the target domain data. Consequently, the model becomes more adept at precisely capturing semantic information within the target domain and learning label knowledge from the source domain, ultimately leading to improved performance in the target domain.

In the future, we will further research and explore how graph information bottleneck theory can select efficient compression strategies in graph adaptation tasks and avoid overfitting in the source domain. And how to avoid the potential amplification of the impact of adversarial attacks on meta pseudo labels during multiple distillation processes. At the same time, we plan to conduct experiments on the domain adaptation task of node-link prediction. We also aim to explore interpretable research to identify the invariant features in the source and target domains and uncover the captured semantic information in the target domain. This deeper understanding and analysis of graph adaptation tasks will facilitate further advancements in the field.

## Supplementary Information

Supplementary Information.

## Data Availability

The data supporting the findings of this study are available within the manuscript or supplementary information files.
