# Supplementary material for: TO-UGDA: target-oriented unsupervised graph domain adaptation
Source: Sci Rep. 2024 Apr 22;14:9165. doi: 10.1038/s41598-024-59890-y (PMC11576983; doi:10.1038/s41598-024-59890-y)
Supplement: Supplementary file 1 — Supplementary Information. [file 41598_2024_59890_MOESM1_ESM.pdf]

Supplementary Appendix

A. Variational upper bound of mutual information constraint

The graph information bottleneck terms $I^S(X_s;Z_s) \leq \gamma$ and $I^T(X_t;Z_t) \leq \gamma$ in Eq. (5) are synchronous to constraint about graph representation from two domains by same GIB constraint module $I(X;Z)$, but the computation of mutual information becomes challenging when dealing with high-dimensional random variables[63]. So in this section, the variational upper bound $I^{up}(X;Z)$ is derived to calculate the information bottleneck constraint $I(X;Z)$ about input X and representation Z.

The mutual information constraint module is to obtain a feature encoder F_θ with parameter θ, which can extract latent invariant feature representation Z in the graph data G via invariant sub-information G_{sub}. $F_\theta(G)$ essentially gives a invariant information distribution $\mathbb{P}_\theta(G_{sub}|G)$ in the graph feature space, so the constraint $I(X;Z)$ can be expressed by the exact definition of mutual information, as follows:

$$I(X;Z) = I(G;Z) = I(G;G_{sub}) = \sum_{G_{sub},G} \mathbb{P}_\theta(G_{sub},G)\log\frac{\mathbb{P}_\theta(G_{sub} \mid G)}{\mathbb{P}(G_{sub})} = \mathbb{E}_{G_{sub},G}\left[\log\frac{\mathbb{P}_\theta(G_{sub} \mid G)}{\mathbb{P}(G_{sub})}\right] \tag{14}$$

where $\mathbb{E}$ denotes the statistical expectation. However, the representation distribution $\mathbb{P}(G_{sub}) = \int \mathbb{P}_\theta(G_{sub}|G)\mathbb{P}_G(G)$ is intractable, we use a variational approximation $\mathbb{Q}(G_{sub})$ of $\mathbb{P}(G_{sub})$ to obtain a variational upper bound of mutual information constraint:

$$\begin{aligned} I(G;G_{sub}) &= \mathbb{E}_{G_{sub},G}\left[\log\frac{\mathbb{P}_\theta(G_{sub} \mid G)}{\mathbb{P}(G_{sub})}\right] = \mathbb{E}_{G_{sub},G}\left[\log\frac{\mathbb{P}_\theta(G_{sub} \mid G)\mathbb{Q}(G_{sub})}{\mathbb{Q}(G_{sub})\mathbb{P}(G_{sub})}\right] \\ &= \mathbb{E}_{G_{sub},G}\left[\log\frac{\mathbb{P}_\theta(G_{sub} \mid G)}{\mathbb{Q}(G_{sub})} - \log\frac{\mathbb{P}(G_{sub})}{\mathbb{Q}(G_{sub})}\right] = \mathbb{E}_{G_{sub},G}\left[\log\frac{\mathbb{P}_\theta(G_{sub} \mid G)}{\mathbb{Q}(G_{sub})}\right] - KL(\mathbb{P}(G_{sub})\,\|\mathbb{Q}(G_{sub})) \\ &\leq \mathbb{E}_{G_{sub},G}\left[\log\frac{\mathbb{P}_\theta(G_{sub} \mid G)}{\mathbb{Q}(G_{sub})}\right] = \mathbb{E}_G\left[KL(\mathbb{P}_\theta(G_{sub} \mid G)\,\|\mathbb{Q}(G_{sub}))\right] \triangleq I_\theta^{up}(X;Z) \end{aligned} \tag{15}$$

By applying the variational upper bound $I_\theta^{up}(X;Z)$ of double GIBs information constraint in Eq. (5), the feature extractor F_θ can filter task-irrelevant information and learn the minimal sufficient features for unsupervised graph adaptation learning.

B. GIB-based adversarial adaptation optimization

To evaluate the mutual information within the optimization objectives $I(Z_s;Y_s) + I(Z_s;Z_t)$ in Eq. (5), we can utilize the equivalent terms to train the adaptive model $M = F \circ C$ comprising a feature extractor F and a classifier C.

B1. Variational lower bound of feature-label mutual information

In the first term in Eq. (5), maximizing the relevance $I(Z_s;Y_s)$ between the latent representations Z and the ground truth labels Y can be expressed by mutual information definition:

$$I(Z_s;Y_s) = I(G;Y_s) \overset{iid}{=} I(G_{sub};Y_s) = \mathbb{E}_{G_{sub},Y_s}\left[\log\frac{\mathbb{P}(Y_s \mid G_{sub})}{\mathbb{P}(Y_s)}\right] = \mathbb{E}_{G_{sub},Y_s}\left[KL(\mathbb{P}(Y_s \mid G_{sub})\,\|\mathbb{P}(Y_s))\right] \tag{16}$$

where $\mathbb{P}(Y_s \mid G_{sub})$ is intractable. If a variational approximation $\mathbb{P}_\theta(Y_s \mid G_{sub})$ is introduced, the lower bound $I^{low}(G_{sub};Y_s)$ can be obtained:

$$\begin{aligned} I(G_{sub};Y_s) &= \mathbb{E}_{G_{sub},Y_s}\left[\log\frac{\mathbb{P}(Y_s \mid G_{sub})\mathbb{P}_\theta(Y|G_{sub})}{\mathbb{P}(Y_s)\mathbb{P}_\theta(Y_s|G_{sub})}\right] = \mathbb{E}_{G_{sub},Y_s}\left[\log\frac{\mathbb{P}_\theta(Y_s|G_{sub})}{\mathbb{P}(Y_s)} + \log\frac{\mathbb{P}_\theta(Y_s|G_{sub})}{\mathbb{P}(Y_s|G_{sub})}\right] \\ &= \mathbb{E}_{G_{sub},Y_s}\left[\log\frac{\mathbb{P}_\theta(Y_s \mid G_{sub})}{\mathbb{P}(Y_s)}\right] + \mathbb{E}_{G_{sub}}\left[KL(\mathbb{P}(Y_s|G_{sub})\,\|\mathbb{P}_\theta(Y_s|G_{sub}))\right] \\ &\geq \mathbb{E}_{G_{sub},Y_s}\left[\log\frac{\mathbb{P}_\theta(Y_s \mid G_{sub})}{\mathbb{P}(Y_s)}\right] = \mathbb{E}_{G_{sub},Y_s}\left[\log\mathbb{P}_\theta(Y_s \mid G_{sub})\right] + H(Y_s) \triangleq I^{low}(G_{sub};Y_s) \end{aligned} \tag{17}$$

where $H(Y_s)$ is the information entropy of label Y_s. So we can maximize $I(Z_s;Y_s)$ by maximizing the lower bound $I^{low}(G_{sub};Y_s)$.

B2. Equivalent substitution about maximizing feature-label mutual information

Maximizing the lower bound $I^{low}(G_{sub};Y_s)$ of $I(Z_s;Y_s)$, which can be equivalently achieved by minimizing the classification loss $\mathscr{L}_{cla}$ of the adaptive model M, as follows:

$$\begin{aligned}\max I(Z_s;Y_s) = \max I(G_{sub};Y_s) &= \max \mathbb{E}_{G_{sub},Y_s}\left[\log \mathbb{P}_\theta\left(Y_s \mid G_{sub}\right)\right] + H(Y_s)\\ &= \max KL\left(\mathbb{P}_{data}(Y_s)\,||\mathbb{P}_\theta\left(Y_s \mid G_{sub}\right)\right)\\ &= \min \mathbb{E}_{(x,y)\sim(X_s,Y_s)}[\textit{Cross-Entropy}(C\circ F(x),y)]\\ &= \min \mathscr{L}_{cla}\left(F,C;\theta_f,\theta_c\right)\end{aligned} \tag{18}$$

where θ_f,θ_c are the parameters of both the feature extractor F and the classifier C. The classification training loss function about M can be defined as :

$$\mathscr{L}_{cla}\left(F,C;\theta_f,\theta_c\right) = \mathbb{E}_{(x,y)\sim(X_s,Y_s)}[\textit{Cross-Entropy}(C\circ F(x),y)] \tag{19}$$

where ℓ is the classification error and $\mathbb{E}_{(x,y)\sim(X_s,Y_s)}$ denote the expectation about source domain labeled data.

B3. Equivalent substitution about maximizing domain alignment mutual information

The maximizing $I(Z_s;Z_t)$ to achieve invariant representation alignment and transfer the source label information Y, which can be inferred to a KL-divergence representation by mutual information:

$$I(Z_s;Z_t) = \mathbb{E}_{Z_s,Z_t}\left[\log\frac{\mathbb{P}(Z_s \mid Z_t)}{\mathbb{P}(Z_s)}\right] = \mathbb{E}_{Z_s,Z_t}\left[\log\frac{\mathbb{P}(Z_s,Z_t)}{\mathbb{P}(Z_s)\,\mathbb{P}(Z_t)}\right] = \mathbb{E}_{s,t}\left[KL\Big(\mathbb{P}(Z_s,Z_t)\,||\mathbb{P}(Z_s)\,\mathbb{P}(Z_t)\Big)\right] \tag{20}$$

Because the calculation challenge of mutual information, the *Donsker-Varadhan Representation*[50,51] based on KL divergence provides a lower limit of mutual information, as follows:

$$\begin{aligned}D_{KL}(\,\mathbb{J}\,||\,\mathbb{M}\,) = \sup_{T:\Omega\to\mathbb{R}} \mathbb{E}_{\mathbb{J}}[T] - \log(\mathbb{E}_{\mathbb{M}}[e^T]) &\geq \sup_{T\in\mathscr{F}} \mathbb{E}_{\mathbb{J}}[T] - \log(\mathbb{E}_{\mathbb{M}}[e^T])\\ &= \mathbb{E}_{\mathbb{J}}[D_\theta(z_s,z_t)] - \log(\mathbb{E}_{\mathbb{M}}[e^{D_\theta(z_s,z_t)}])\\ &\triangleq I_\theta^{DV}(Z_s;Z_t)\\ &= I_\theta^{DV}\left(F(X_s),F(X_t)\right)\end{aligned} \tag{21}$$

where $\mathbb{J}$ represents the joint distribution $\mathbb{P}(Z_s,Z_t)$, and the $\mathbb{M}$ represents the product $\mathbb{P}(Z_s)\,\mathbb{P}(Z_t)$ of marginal distributions about (Z_s,Z_t). $\mathscr{F}$ is any class of function $T:\Omega\to\mathbb{R}$ satisfying the integrability constraints of the *Donsker-Varadhan Representation*. D_θ is an instance of functions $\mathscr{F}$ by a deep neural network with parameter $\theta\in\Theta$.

Maximizing mutual information $I(Z_s;Z_t)$ between the latent representations Z_s and Z_t of the source and target domains, which can be equivalently achieved by adversarial alignment $\max \mathscr{L}_{adv}\left(F,D;\theta_f,\theta_d\right)$ based on maximizing the lower bound *Donsker-Varadhan Representation* $\max I_\theta^{DV}\left(F(x_s),F(x_t)\right)$ about feature encoder F, as follows:

$$\begin{aligned}\max I(Z_s;Z_t) &= \max I_\theta^{DV}\left(F(x_s),F(x_t)\right)\\ &= \max \mathbb{E}_{\mathbb{J}}[D_{\theta_d}\left(F_{\theta_f}(x_s),F_{\theta_f}(x_t)\right)] - \log(\mathbb{E}_{\mathbb{M}}[e^{D_{\theta_d}\left(F_{\theta_f}(x_s),F_{\theta_f}(x_t)\right)}])\\ &= \max \mathscr{L}_{adv}\left(F,D;\theta_f,\theta_d\right)\end{aligned} \tag{22}$$

where, the maximizing mutual information $I(Z_s;Z_t)$ can be equivalently achieved by the adversarial alignment to align the latent spaces of the domains, as follows:

$$\mathscr{L}_{adv}(F,D;\theta_f,\theta_d) = \mathbb{E}_{x\sim X_s}[D(F(x))] + \mathbb{E}_{x\sim X_t}[1-D(F(x))] \tag{23}$$

where F is encouraged to reduce the discrepancy of feature distribution by confusing the domain discriminator D.

B4. The optimization based on GIB framework

Finally, each mutual information term in Eq.(5) can be efficiently calculated, and meets the task conditions of utilizing the graph information bottleneck theory[22]:

$$\min_{\mathbb{P}(Z|X)\in\Omega} GIB(X,Y;Z) \triangleq \min_{\theta_{f,c}} \left[-I(Z;Y)+\beta I(X;Z)\right] \tag{24}$$

where β is the weight factor, Z represents the latent variable that contains irrelevant information, and Ω denotes the search space of the optimal representation model $\mathbb{P}(Z|X) \sim \mathbb{P}(G_{sub}|G)$ about graph data X. It also has an equivalent form:

$$\begin{aligned} &\max_{\mathbb{P}(Z|X)\in\Omega} I(Z;Y) \\ &\text{s.t. } I(X;Z) \leq \gamma \end{aligned} \tag{25}$$

Intuitively, Eq. (24) and Eq. (25) encourage the representation Z try their best to capture the information in Y, while using information constraints $I(X;Z)$ to avoid the interference of excessive irrelevant information.

Using the aforementioned equivalent substitution, multiple mutual information terms in Eq. (5) can be cleverly replaced with other objective functions that are easier to solve. By leveraging Eq. (24), the graph adaptive task can be transformed into the GIB paradigm. Therefore, the resulting equivalent adaptation objective about Eq. (5) is as follows:

$$\mathscr{L}_{\text{adapt}}(F,C,D) = \min_{F,C}\max_{D}\left(\mathscr{L}_{\text{cla}}(F,C;\theta_{f,c}) + \mathscr{L}_{\text{adv}}(F,D;\theta_{f,d}) + \beta\left(I^{\text{up}}_{\theta_f}(X_s;Z_s) + I^{\text{up}}_{\theta_f}(X_s;Z_t)\right)\right) \tag{26}$$

C. Unsupervised meta pseudo labels distillation

C1. The updating direction of teacher model

Due to the student model θ_S rely on the target domain pseudo label generated by teacher model θ_T in Eq. (9), making distillation impossible to directly update the parameters of teacher model by the performance of student model. Therefore, we employ a step-by-step update approach for both parameters to alternate the updates of θ_T and θ_S.

Since the current optimal solution only represents the local optimal of the objective function, we adopted the stepwise alternate updating method to replace directly updating to the optimal value of the current model, aiming to improve the global optimal solution. In the distillation loop, one model is updated by taking only one step along the gradient direction of the model, while other model remains fixed. Therefore, we adjust θ_S by the target domain pseudo label:

$$\theta_S' \approx \theta_S - \eta_S \cdot \nabla_{\theta_S}\mathscr{L}_{\text{adapt}}\left(S([x_s,x_t];\theta_S),\hat{y}_t\right) \tag{27}$$

the teacher model θ_T is updated based on the adaptation gradient and the performance feedback $\mathscr{L}_{\text{distill}}$ of updated student model θ_S':

$$\theta_T' = \theta_T - \eta_T \cdot \nabla_{\theta_T}\mathscr{L}_{\text{adapt}}\left(S([x_s,x_t];\theta_T),y_s\right) - \eta_T \cdot \nabla_{\theta_T}\mathscr{L}_{\text{distill}}(T,S;[x_s,x_t,y_s],\theta_S'(\theta_T)) \tag{28}$$

where the derivative of the teacher distillation gradient $\nabla_{\theta_T}\mathscr{L}_{\text{distill}}$ about student performance feedback gradient ∇_{θ_S} and the dependency relationship $\nabla_{\theta_T}\theta_S'(\theta_T)$ can be expressed as the product of two derivatives to calculate:

$$\frac{\partial\mathscr{L}_{\text{distill}}}{\partial\theta_T} = \frac{\partial\mathscr{L}_{\text{distill}}}{\partial\theta_S'(\theta_T)} \cdot \frac{\partial\theta_S'(\theta_T)}{\partial\theta_T} = d \cdot \frac{\partial\mathscr{L}_{\text{adapt}}\left(T([x_s,x_t];\theta_T),\hat{y}_t\right)}{\partial\theta_T} \tag{29}$$

where $\hat{y}_t$ represents the soft pseudo-labels, and $d = \left(\mathscr{L}_{\text{cla}}(S(x_t;\theta_S),y_s) - \mathscr{L}_{\text{cla}}(S(x_s;\theta_S'),y_s)\right)$ is obtained using Taylor's Formula in meta pseudo-label learning[24], whose difference is computed on the labeled source domain validation dataset by student model after updating. The last term $\frac{\partial\mathscr{L}_{\text{adapt}}\left(T([x_s,x_t];\theta_T),\hat{y}_t\right)}{\partial\theta_T}$ in Eq. (29) represents the loss gradient between the teacher network output and the pseudo-labels for the adaptation task.

C2. Training algorithm of TO-UGDA

Algorithm 1: Training algorithm of TO-UGDA

input : Source domain dataset $\mathscr{D}^s$, Target domain dataset $\mathscr{D}^t$, Updating steps N

output : GNN parameters θ_e, Classifier parameters θ_f

Initialize the GNN parameters for framework by joint pre-training.;

for $n \leftarrow 1$ **to** N **do**

/* GIB-based domain adversarial adaptation */;

Compute classification loss $\mathscr{L}_{\text{cla}}\left(T\left(x_t;\theta_S\right), y_s\right)$ of teacher model via Eq. (19) ;

Compute adaptation loss $\mathscr{L}_{\text{adv}}\left(x_t, x_s, y_s;\theta_S\right)$ of teacher model via Eq. (23) ;

Compute mutual information constraint bound $I^{\text{up}}\left(X_s;Z_s\right)$) and $I^{\text{up}}\left(X_t;Z_t\right)$) of teacher model via Eq. (15);

Compute adaptation loss $\mathscr{L}_{\text{adapt}}\left(T\left(\left[x_s, x_t\right];\theta_T\right), y_s\right)$ of teacher model via Eq. (8);

/* Meta pseudo label knowledge distillation */;

Generate Pseudo-Label $\left(x_t, \hat{y}_t\right)$ of target domain data via teacher model;

Compute adaptation loss $\mathscr{L}_{\text{adapt}}\left(T\left(\left[x_s, x_t\right];\theta_T\right), \hat{y}_t\right)$ of teacher model via Eq. (8);

Compute loss $\mathscr{L}_{\text{cla}}\left(S\left(x_s;\theta_S\right), y_s\right)$ of semantic clustering student model via Eq. (19);

Compute adaptation loss $\mathscr{L}_{\text{adapt}}\left(S\left(\left[x_s, x_t\right];\theta_S\right), \hat{y}_t\right)$ of student model via Eq. (8);

Update parameters $\theta_S^{'}$ of student model for $F, C, D.$ via Eq. (27);

Compute loss $\mathscr{L}_{\text{cla}}\left(S\left(x_s;\theta_S^{'}\right), y_s\right)$ of student model via Eq. (19);

Compute bargaining distillation loss $\mathscr{L}_{\text{distill}}\left(\theta_T, \theta_S\right)$ via Eq. (12);

Update parameters $\theta_T^{(n)}$ of teacher model for F, C, D via Eq. (13);

end

return Teacher model parameters $\theta_T^{(n)}$ and Student model parameters $\theta_S^{(n)}$;
